# Health involvement modulates physician preference in the brain during online health consultation

**DOI:** 10.1038/s41598-024-51519-4

**Published:** 2024-01-13

**Authors:** Yifan Zhang, Yan Wan, Hengyi Rao

**Affiliations:** 1 School of Modern Posts, Chongqing University of Posts and Telecommunications, Chongqing, 400065 China; 2 School of Economics and Management, Beijing University of Posts and Telecommunications, Beijing, 100876 China; 3 Center for Magnetic Resonance Imaging Research & Key Laboratory of Applied Brain and Cognitive Sciences, School of Business and Management, Shanghai International Studies University, Shanghai, 200083 China; 4 Department of Neurology, Perelman School of Medicine, Center for Functional Neuroimaging, University of Pennsylvania, Philadelphia, PA 19104 USA

**Keywords:** Cognitive neuroscience, Human behaviour

## Abstract

In traditional offline health-seeking behavior, patients consistently exhibit a preference for similar types of physicians due to limited access to physicians’ information. Nevertheless, with the advent of online health consultation platforms offering comprehensive physicians’ information for patients, raises the question: do patients continue to exhibit uniform preference for physicians? To address this issue, we first employed a behavioral experiment to discern patients’ preferences for different types of physicians’ information under different health involvement, and then conducted a functional magnetic resonance imaging (fMRI) experiment to furnish neural/physiological evidence. The results showed that health involvement modulates patients’ preferences, when health involvement was low, patients had diverse preferences for physicians, that is, different types of physicians’ information could individually impact patients’ choice and could serve as substitutes for each other. When health involvement was high, patients’ preference for physicians were uniform, highlighting that the collective influence of different types of physicians’ information on patients’ choice behavior. From the neural level, an explanation for the results was that the ventromedial prefrontal cortex (VMPFC) and ventral striatum (VS) brain regions, two key brain regions reflecting individual cognitive resource allocation, had different activation levels under different health involvement, indicating that patients allocated different cognitive resources.

## Introduction

Online health consultation is a novel form of doctor-patient communication that has emerged alongside the rapid advancements in Internet technology^1^. Patients can employ mobile phones, computers and other terminal devices to engage in various communication modes, such as pictures, texts, voice or phone consultation on online health consultation platforms. Similarly, physicians can also choose an appropriate method to provide professional advice for patients^2^. In traditional offline health-seeking behavior, patients face limitations in accessing physicians’ information when choosing a healthcare provider^3^, because beyond readily identifiable information such as the hospital and professional title of physicians, obtaining nuanced information concerning personal characteristics and communication styles of physicians poses a frequent obstacle for patients. While online health consultation platforms can provide a wealth of information, such as physicians’ professional title, service attitude, communication styles, response speed and so on. This multifaceted information help patients form cognitive judgments and affective attitudes towards a physician, so as to make further decisions^4,5^.

As mentioned above, in traditional offline health-seeking behavior, patients have limited access to physicians’ information^3^. This leads to a scenario where patients’ preferences for physicians tend to converge, resulting in a chaotic pursuit of top-tier hospital renowned physicians irrespective of major or minor ailments^6^. So now online health consultation platforms can provide comprehensive physicians’ information for patients^4,7^, do patients still exhibit uniform preference for physicians? That is, what specific type of physician information do patients rely on to make decisions when choosing a physician on online health consultation platform? Do they continue to prioritize cognitive stimulation information that can reflect the physician’s professionalism, such as the hospital and professional title of physicians^2^, or do they favor affective stimulation information, which reflects the physician’s care for the patient, such as the physician's attitude and communication skills^2^? Thus, the primary objective of this study is to explore the preferences of patients for different types of physician information during the process of choosing physicians on online health consultation platforms.

Previous studies have shown that the influence of information on individual decision-making behavior is regulated by environmental and contextual factors. These factors encompass the characteristics of users, products or services^8^, of which involvement is an important variable that modulates this influence relationship^9–11^. Involvement is originally a psychological concept, referring to the perceived significance and relevance that individuals attribute to specific entities^12^. Diverse manifestations of involvement emerge based on the objects under consideration, including but not limited to product involvement, shopping involvement, brand involvement and advertising involvement^13^. In the field of health decision-making, the importance and relevance of different disease types to patients exhibit distinctions. Therefore, this study defines different disease types as health involvement^14^. Results from previous studies indicate that patients’ behavior is modulated by health involvement, and patients with different disease types exhibit distinct preferences for physicians’ information. For example, Lu and Wu^8^ found that the lower the risk of disease, that is, the lower the health involvement, patients’ choice behavior becomes more susceptible to affective stimulus information, such as physicians’ attitude. Thus the first objective of this study is to investigate the moderating effect of health involvement (disease type) on the patients’ preferences for physicians’ information on online health consultation platforms.

However, it is evident that the same disease varying degrees of significance among distinct patients. For example, individuals deeply preoccupied with their health status or even have health anxiety^15^, they may perceive a common ailment such as a headache as a potentially grave threat to their well-being. This perception could subsequently influence their health information searching behavior and even health decision-making^16,17^. Conversely, individuals with lower levels of health anxiety may regard the same symptom as less severe, deeming it necessitating only minor recuperation. Consequently, during the follow-up experiments, deliberate efforts were made to minimize the potential influence of health anxiety on the experimental conditions pertaining to health involvement.

Individual preference or adoption of different types of information is actually a process in which an individual makes a value evaluation on the received information and then makes decision according to the value ranking of different alternatives^18–20^. Hence, the neural mechanism of individual information value ranking process can refer to value-based decision theory. Value-based decision theory posits that the varied preferences and attitudes of individuals towards the same entity are termed subjective value. Subjective value enables individuals to integrate diverse and complex alternatives into a common dimension for comparative evaluation^21,22^. On the neural level, extensive research has shown that the ventromedial prefrontal cortex (VMPFC) and ventral striatum (VS) are crucial brain regions associated with subjective value. These regions exhibit notable activation during individual evaluations of subjective value^20–23^. Therefore, the second objective of this study is to reveal the neural mechanism of the moderating effect of health involvement on patients’ preferences for physicians’ information by analyzing the activation differences of VMPFC and VS brain regions when patients process information under different health involvement.

According to the above, to achieve our two objectives in this paper, we first employed behavioral experiment to identify patients’ preferences for different types of physicians’ information (cognitive stimulation information and affective stimulation information) under different health involvement, and then used functional magnetic resonance imaging (fMRI) experiment to analyze the brain activation mechanism of patients under different health involvement from the neural level, thereby providing neural/physiological evidence elucidating the influence of health involvement on the information preferences of patients in online health consultation services.

## Results

### Manipulation check results

Through the manipulation checks, this paper selected four types of physician information from the seven types of physician information extracted on the online health consultation platform for formal experiment. The cognitive stimulation category included: physician rank (p < 0.001) and professional knowledge (p < 0.001), while the affective stimulation category encompassed service attitude (p = 0.012) and communication skills (p = 0.033). The results are shown in Table 1.

**Table 1:** Physician information manipulation checks results.

Physician information		CT	Sig	AT	Sig
		Mean (S.D.)		Mean (S.D.)	
Physician rank	100% favorable rating	7.372 (0.732)	0.000	6.769 (0.992)	0.506
	75% favorable rating	6.444 (0.716)		6.602 (0.824)	
Professional knowledge	100% favorable rating	7.513 (0.855)	0.000	6.769 (1.086)	0.973
	75% favorable rating	6.679 (0.754)		6.778 (0.670)	
Treatment effect	100% favorable rating	7.641 (1.045)	0.000	7.346 (0.718)	0.022
	75% favorable rating	6.531 (0.921)		6.889 (0.695)	
Service attitude	100% favorable rating	7.115 (0.699)	0.127	7.327 (0.897)	0.012
	75% favorable rating	6.840 (0.595)		6.750 (0.714)	
Communication skills	100% favorable rating	7.000 (0.993)	0.321	7.212 (0.836)	0.033
	75% favorable rating	6.753 (0.793)		6.639 (1.050)	
Response speed	100% favorable rating	7.090 (0.910)	0.072	7.038 (0.734)	0.300
	75% favorable rating	6.716 (0.478)		6.843 (0.625)	
Service commitment	100% favorable rating	7.205 (1.360)	0.087	7.231 (1.317)	0.105
	75% favorable rating	6.593 (1.192)		6.778 (0.543)	

Note: CT, cognitive trust; AT, affective trust; all the cognitive and affective trust scores ranged between 1 to 9.

### Behavioral results

This paper used 2 (cognitive trust high/low) × 2 (affective trust high/low) behavioral experiment to explore the influence of cognitive trust and affective trust on patients’ willingness to choose under two different levels of health involvement (high/low) through two-way repeated ANOVAs, so as to identify patients’ preference to cognitive and affective stimulation information under different levels of health involvement. The results showed that health involvement had a significant impact on patients’ preferences for different types of physicians’ information. When health involvement was low, the interaction between cognitive trust and affective trust on participants’ willingness to choose was not significant (df = 47, F = 0.323, p = 0.572; Partial η^2^ = 0.007). Conversely, under conditions of high health involvement, the interaction between cognitive trust and affective trust on participants’ willingness to choose was significant (df = 45, F = 13.049, p = 0.001; Partial η^2^ = 0.225). The results are shown in Table 2.

**Table 2:** Two-way multivariate ANOVA results of patients’ scores of willingness to choose with different levels of health involvement.

Health involvement	Source of variance	df	F	Sig	Partial η^2^
Low level health involvement	CT	1	22.528	0.000	0.324
	AT	1	31.853	0.000	0.404
	CT*AT	1	0.323	0.572	0.007
	Error	47			
High level health involvement	CT	1	28.389	0.000	0.387
	AT	1	25.968	0.000	0.366
	CT*AT	1	13.049	0.001	0.225
	Error	45			

Note: CT, cognitive trust; AT, affective trust.

**Figure 1:**
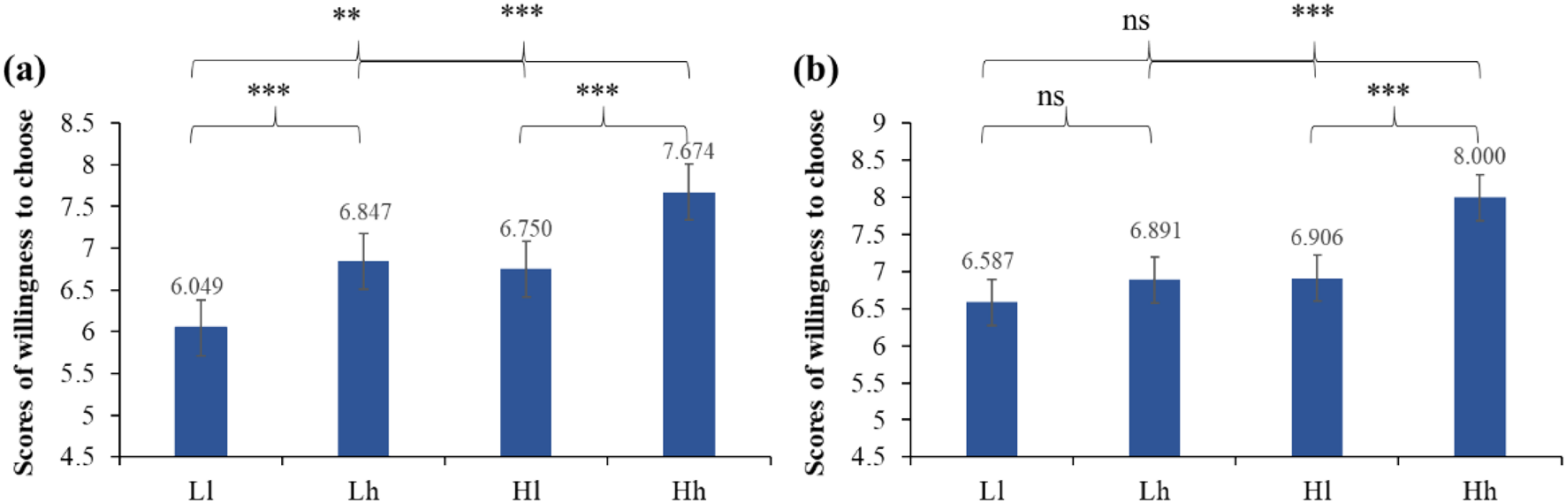
Simple effect analysis results of patients' scores of willingness to choose with different levels of health involvement. Note: (**a**) low level health involvement; (**b**) high level health involvement; H, high level of cognitive trust; L, low level of cognitive trust; h, high level of affective trust; l, low level of affective trust; all the scores of willingness to choose ranged between 1 and 9; ns p > 0.05, *p <  = 0.05, **p <  = 0.01, ***p <  = 0.001; error bars: SEM.

Further post-hoc test found that (see Table 3 and Fig. 1), when the health involvement was low, both cognitive and affective stimulus information could influence patients' decision-making behavior. The results indicated that an enhancement in either cognitive trust or affective trust significantly heightened patients’ willingness to choose the physician. When health involvement was high, the isolated improvement of either cognitive stimulus information or affective stimulus information did not elicit any discernible impact on patients' decision-making behavior. The outcomes illustrated that enhancing either cognitive trust or affective trust independently did not significantly improve participants’ willingness to choose. Notably, it was only when both aspects were improved simultaneously that participants’ willingness to choose would be significantly improved.

**Table 3:** Simple effect analysis results of patients’ scores of willingness to choose with different levels of health involvement.

Health involvement	Variance		Mean (S.D.)	95% confidence interval for difference		Sig
				2.5%	97.5%	
Low level health involvement	H	h	7.674 (1.075)	0.578	1.269	0.000
		l	6.750 (1.112)			
	L	h	6.847 (1.138)	0.390	1.207	0.000
		l	6.049 (1.645)			
	h	H	7.674 (1.075)	0.523	1.130	0.000
		L	6.847 (1.138)			
	l	H	6.750 (1.112)	0.237	1.165	0.004
		L	6.049 (1.645)			
High level health involvement	H	h	8.000 (1.035)	0.725	1.463	0.000
		l	6.906 (1.340)			
	L	h	6.891 (1.201)	− 0.033	0.641	0.076
		l	6.587 (1.416)			
	h	H	8.000 (1.035)	0.764	1.453	0.000
		L	6.891 (1.201)			
	l	H	6.906 (1.340)	− 0.033	0.671	0.075
		L	6.587 (1.416)			

Note: H, high level of cognitive trust; L, low level of cognitive trust; h, high level of affective trust; l, low level of affective trust; all the scores of willingness to choose ranged between 1 and 9.

### Imaging results

**Figure 2:**
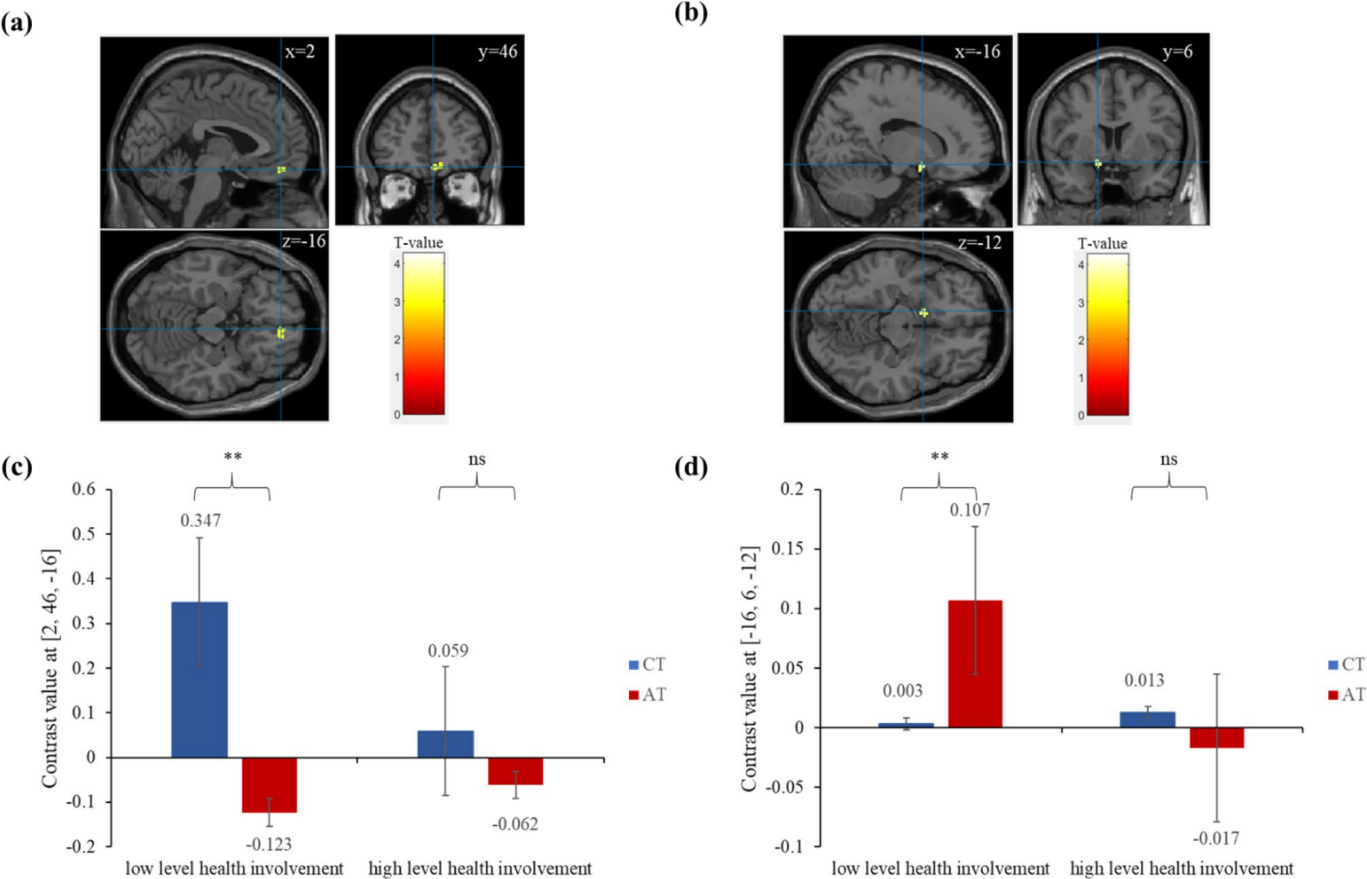
Task-related activation in the VMPFC and VS. Note: (**a**) activation for the CT contrast in the VMPFC with low level health involvement; (**b**) activation for the AT contrast in the VS with low level health involvement; (**c**) plots of contrast values in the VMPFC; (**d**) plots of contrast values in the VS; CT = CT contrast, AT = AT contrast; ns p > 0.05, *p <  = 0.05, **p <  = 0.01, ***p <  = 0.001; error bars: SEM.

To examine the activation differences of VMPFC and VS brain regions when patients process information under different health involvement conditions, this paper first used the general linear model (GLM) in SPM12 to generate the design matrix for the individual-level analyses (Specify 1st-level), calculated the task activation regions under four different experimental conditions of Hh, Hl, Lh, Ll for each participant, and then performed group-level analyses (Specify 2nd-level). On the group-level, we computed the contrast “CT [(Hh + Hl)−(Lh + Ll), AT [(Hh + Lh)−Hl + Ll)]” (for additional experimental parameters and design matrix used in the SPM analysis, see Supplementary 1. Fig. S1). Within our priori ROIs hypotheses-driven, we focused on VMPFC (6-mm sphere centered at the MNI coordinate: 2/46/− 16)^24^ and VS (6-mm sphere centered at the MNI coordinate: − 16/6/− 12)^25^ these two regions of interest (ROIs), extracted the contrast value of these two ROIs to analyze their differences in brain activation under different health involvement conditions. The results are shown in Fig. 2 (for whole-brain results, see Supplementary 1. Table S1), in the condition of low health involvement, activation in the VMPFC differed between CT contrast and AT contrast (t = 3.232, df = 31, p = 0.003; Cohen’s d = 0.572), CT contrast in the VMPFC was significantly activated (small volume FWE corrected at p = 0.001 and k = 26), but not AT contrast. Activation in the VS also differed between CT contrast and AT contrast (t = 2.800, df = 31, p = 0.009; Cohen’s d = 0.498), AT contrast in the VS was significantly activated (small volume FWE corrected at p = 0.003 and k = 17), while CT contrast was not significantly activated. In the condition of high health involvement, activation in the VMPFC (t = 0.762, df = 31, p = 0.452; Cohen’s d = 0.134) and VS (t = 0.699, df = 31, p = 0.489; Cohen’s d = 0.123) was not significantly different between CT contrast and AT contrast, and neither brain region was significantly activated in either contrast.

## Discussion

The results of behavioral experiment indicate that the preferences of patients in choosing physician online varied based on their levels of health involvement. When the health involvement was low, physicians with professionalism or good attitude can be preferred by patients. This study observed a noteworthy correlation between the improvement of cognitive trust or affective trust and patients’ willingness to choose physicians, which means that both cognitive stimulation information and affective stimulation information can influence the patient’s decision-making, and the effects of the two can be mutually substituted/compensated. When the health involvement was high, patients exclusively favored physicians possessing both good professionalism and attitude. This suggests that an increase in either cognitive trust or affective trust alone did not substantially improve patients’ willingness to choose. Instead, a significant improvement in the willingness to choose only occurred when both aspects were enhanced simultaneously. This underscores the crucial role of combining cognitive and affective stimulus information in influencing patients’ choice behavior.

The results have also been confirmed at the neural level. Imaging experiment revealed that patients with low health involvement exhibited significant activation in the VMPFC when evaluating cognitive stimulation information and in the VS when evaluating affective stimulation information, and previous studies have demonstrated the close association of both VMPFC and VS with subjective value evaluation ^20–23^. This implies that when health involvement is low, patients integrate cognitive and affective stimulation information within the same dimension for comparative evaluation to make decisions ^21,22^. In essence, patients with low health involvement exhibit a preference for both cognitive and affective stimuli information. Conversely, when health involvement was high, the results indicated the absence of activation in related brain regions when patients evaluated cognitive and affective stimulation information. According to previous studies, the activation degree of related brain regions can serve as a reflection of cognitive resources allocated by individuals^26^. Therefore, the results of the imaging experiment may provide a theoretical explanation for the modulation of health involvement on patients’ preferences for different types of physicians’ information.

According to the Elaboration Likelihood Model (ELM), a theoretical framework delineating individual information processing, two distinct routes exist for individuals when processing information, one is the central route and the other is the peripheral route^27^. When an individual uses the central route to process information, the individual's attitude will change after a comprehensive evaluation of the detailed information in a cognitive / rational way, which requires more cognitive resources. When an individual adopts the peripheral route, the individual will engage in a less refined processing of received information, allocating fewer cognitive resources^28–30^. This study employed imaging experiment to reveal the information processing processes of patients. The results indicated that when the health involvement was low, patients had a higher activation degree of brain regions, allocated more cognitive resources, which means they preferred to use the central route to process information. When the health involvement was high, patients allocated less cognitive resources and preferred to use peripheral route to process information.

Although the results in this paper indicated that health involvement had an impact on patients' information processing routes, that is, patients with low health involvement used the central route, while those with high health involvement used peripheral route. However, this finding is inconsistent in the field of consumption decision-making. Previous studies have shown that when the product or service is more important to consumers (i.e., high involvement), they allocate more cognitive resources to evaluate information through the central route. Conversely, under low involvement, consumers resort to the peripheral route for information processing^31–33^. We posit that this discrepancy arises due to the distinct nature of health decision-making compared to product purchases. In the field of consumption decision-making, individuals freely choose their information processing route without restricting cognitive resources. However, in the field of health decision-making, the priority of the disease itself affects the patient's energy. When the disease is milder, patients possess more energy to comprehensively and carefully evaluate all obtained physician information when choosing physicians. Therefore, in instances of low health involvement, patients are more inclined to adopt the central route for information processing. Conversely, under high health involvement, the overwhelming nature of "care is chaos" may deprive patients of sufficient time and energy to comprehensively evaluate all information, leading them to rely on the peripheral route for decision-making.

To sum up, this paper first found the impact of health involvement in the field of online health-seeking on patients' physician preferences through behavioral experiment, and then used imaging experiment to record the brain reaction process of patients’ decision-making to reveal the neural mechanism of this impact, provided neural/physiological evidence to support the derived conclusions. The findings expand the application of individual information processing theories in the field of health decision-making, and establish an effective connection between individual psychological function and decision-making behavior. Furthermore, the relevant conclusions can also provide insights to practical applications. From the behavioral results, it can be found that patients exhibiting diverse disease types employ distinct ways in processing physician information, thereby expressing preferences for physicians of different categories. Consequently, in practical applications, the platform has the potential to formulate a physician recommendation mechanism rooted in these observations. This mechanism could tailor recommendations based on the preferences of patients with specific disease types, fostering a targeted and efficacious alignment between patients and physicians. For example, with respect to patients with mild diseases, the platform can not only recommend physicians demonstrating high professional competence but also those general physicians skilled in cultivating affective trust with patients. This strategic approach mitigates the occurrence of a distorted phenomenon wherein all patients converge toward high-quality medical resources, thereby alleviating the strain on such resources and preventing the idle wastage of ordinary medical resources. Moreover, this approach bolsters the pivotal role of online health consultation platforms in the equitable distribution of doctors and efficient channeling of patient flows. Consequently, it contributes to the optimization of grassroots resource utilization and facilitates the judicious allocation of medical resources.

The study has certain limitations. Firstly, all the experimental methods employed in this study were situational experiments. While situational experiments manage interference from irrelevant variables, a disparity exists between the experimental setting and the real-world scenario. Therefore, future research could employ field experiments or integrate virtual reality technology to enhance the fidelity of the experimental setting, bridging the gap between the experimental and real-world scenarios for more precise results. Secondly, as mentioned earlier, health anxiety can influence patients’ perception of health involvement. Although in the behavioral experiment, participants were categorized into disease types corresponding to their perception of the severity of their previous consultation illness, distinguishing between mild and acute cases. In the imaging experiment, we chose two clearly distinguishable diseases, cold and acute abdominal pain, to represent conditions with low and high health involvement, thereby mitigating potential influences. However, the control of health anxiety constitutes a limitation of this study. Future research should contemplate measuring participants’ health anxiety during the experiment to control this impact. In addition, the participants of the imaging experiment are mainly university students. Despite the predominant users of the online health consultation platform being young people^34^, the significant health demands of the aging population must not be overlooked.. To enhance the effective utilization of online health consultation services among the elderly, future research should prioritize investigating the behavior patterns of this demographic.

## Methods

### Manipulation check

To divide the abundant physician information provided on the online health consultation platform into two types: cognitive stimulation information and affective stimulation information, this paper referred to the physician-related information provided on an actual online health consultation platform, and extracted seven types of physician attributes: (1) physician rank (indicating both the hospital rank and the physician’s professional title); (2) professional knowledge (signifying the specific expertise of the physician); (3) treatment effect; (4) service attitude; (5) communication skills; (6) response speed; (7) service commitment (the presence of physician on the online health consultation platform means that the physician promises to provide services for users online. The indicator of the number of replies in recent two weeks on the actual platform can reflect whether physicians have enough time and energy to provide services for users online, so we use service commitment to represent the number of replies in recent two weeks). 53 participants (M_age_ = 28.98 years) were recruited via the sample service provided by the website wjx (https://www.wjx.cn/). The participants were stratified into two groups: the high satisfaction group (experimental condition: physician information + 100% favorable rating, N = 26) and the low satisfaction group (experimental condition: physician information + 75% favorable rating, N = 27) to assess the seven physician attributes mentioned above. Participants were required to assess both cognitive and affective trust perceptions after reviewing a physician’s information. If the information about a physician differed solely in the cognitive trust scores of the participants, without affecting affective trust, it categorizes as cognitive stimulation information; otherwise it is considered affective stimulation information (the measurement scales see Supplementary 1. Table S2).

### Behavioral experiment

Given the objective of this study is to identify the physician preferences of patients with different health involvement. 94 participants with prior experience in online health consultations were recruited for a mixed-design experiment using the sample service provided by the website wjx (https://www.wjx.cn/). The participants were required to engage in this experiment by recalling their most recent online health consultation experience. Subsequently, participants were categorized into disease types corresponding to their perception of the severity of their previous consultation illness, distinguishing between mild and acute cases. Specifically, 48 participants were low health involvement in mild diseases, 46 participants were high health involvement in acute diseases. The mean age of the participants was 29.28 ± 7.84 years. During the experiment, participants rated the physician’s willingness to choose according to a combination of a cognitive stimulation information + favorite rating and an affective stimulation information + favorite rating. These sets of information were randomly drawn from the physician’s information selected from the manipulation check. Each participant encountered scenarios involving high cognitive trust & high affective trust (Hh: cognitive stimulation information + 100% favorable rating & affective stimulation information + 100% favorable rating), high cognitive trust & low affective trust (Hl: cognitive stimulation information + 100% favorable rating & affective stimulation information + 75% favorable rating), low cognitive trust & high affective trust (Lh: cognitive stimulation information + 75% favorable rating & affective stimulation information + 100% favorable rating), low cognitive trust & low affective trust (Ll: cognitive stimulation information + 75% favorable rating & affective stimulation information + 75% favorable rating) these four experimental conditions, corresponding to four different physicians. Participants were explicitly informed that the four physicians shared identical conditions, differing only in the ratings assigned to the experimental materials. Participants were then instructed to evaluate the willingness to choose for each physician based on the information provided by the experimental materials (the measurement scales see Supplementary 1. Table S2).

### Imaging experiment

The experimental task in the imaging experiment was consistent with the behavioral experimental task. However, to generate a stronger BOLD signal in the imaging experiment, the low satisfaction rating was adjusted from 75% favorable rating to 25% favorable rating. The experimental paradigm employed in this study was rapid-presentation (jittered) event-related design, and the order and timing of events were generated with the help of optseq2 tool^35^. We recruited 33 undergraduate and graduate students as participants for a within-subject design experiment via online promotional channels, including WeChat groups and campus forums. One participant was excluded due to exceeding the specified head movement range of 2 mm, other 32 participants were included in the final analysis (M_age_ = 23.97 ± 2.74 years). All the participants were right-handed, physically healthy, devoid of mental related diseases, and exhibited either normal or corrected vision. Upon completion of the experiment, each participant got RMB 150 yuan as a reward for their participation.

### Scanning procedure

**Figure 3:**
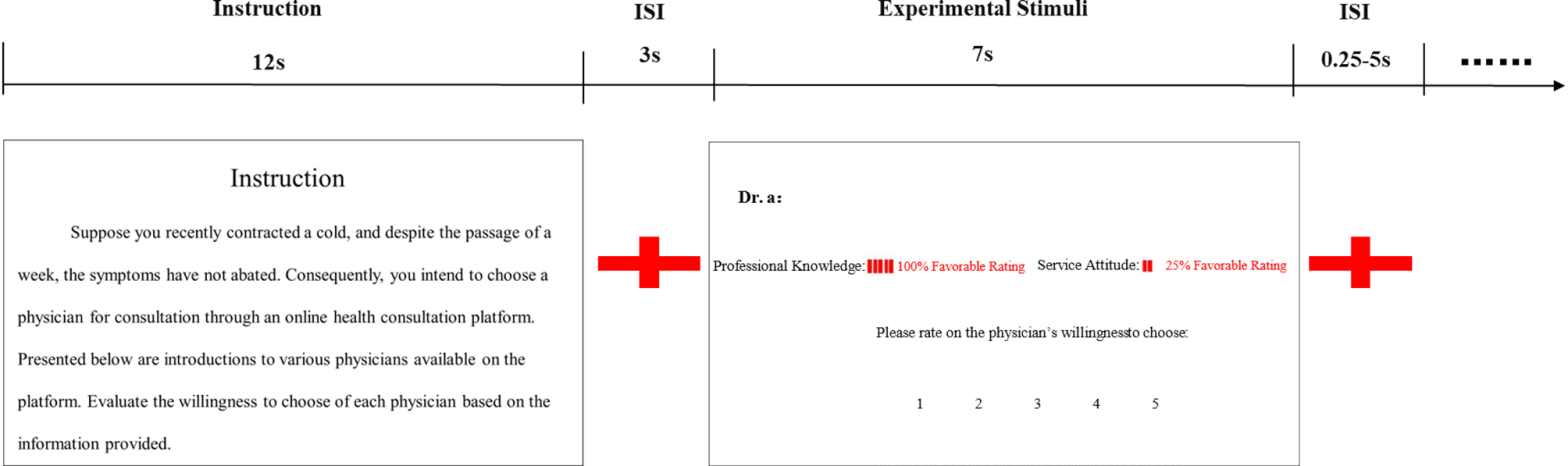
Illustration of trial procedures. Note: ISI, inter-stimulus interval.

The participants first closed their eyes to receive a T1 structural image scan with a duration of about 6 min, and then performed two functional image scans with a duration of about 11 min. Participants were allowed to take a brief respite between each scan at their discretion. The two functional image scans were two tasks, corresponding to the two types of diseases with different health involvement. The imaging experiment was a simulation in which participants were required to hypothetically possess a specific disease. Drawing upon the severity and urgency of the disease, and building on the findings of Li et al.^36^, this study explicitly defined the disease under low health involvement experimental conditions as a cold, while designating acute abdominal pain for high health involvement conditions. In each task, participants were initially presented with an experimental instruction with a duration of 12 s, followed by a fixation point with a duration of 3 s, and then a formal experimental stimulus with a duration of 7 s. Each task contained 80 trails, including four experimental conditions: Hh, Hl, Lh, and Ll. Each condition repeated 20 times within a single task. Participants were instructed to provide keystroke responses during the duration of the formal experimental stimulus. Because the right-handed single-handed keypresses were used, the participants used the Likert 5-level scale to evaluate the physician’s willingness to choose in response to the experimental stimulus (where 1 indicated very unwilling, 5 indicated very willing). The experimental stimulus presentation program is E-prime, and the presentation procedures is shown in Fig. 3.

### Data collection

The imaging data was collected on a Siemens PRISMA 3T fMRI scanner at magnetic resonance imaging research center of Peking University. The scanning parameters of T1 structure images were: Voxel size = 0.5 mm × 0.5 mm × 1.0 m, Field of View = 256 mm, Slice thickness = 1.00 mm, TR = 2530.0 ms, TE = 2.98 ms, Flip angle = 7°, Matrix = 256 × 256. The functional image was scanned by echo planar imaging (EPI), and the scanning parameters were: Voxel size = 2.0 mm × 2.0 mm × 2.0 mm, Slices = 62, Field of View = 224 mm, Slice thickness = 2.0 mm, TR = 2000 ms, TE = 30.0 ms, Flip angle = 90, Matrix = 112 × 112.

### Ethics approval and consent to participate

All study procedures and methods were performed in accordance with the relevant guidelines and regulations and approved by Ethical Review Board of School of Psychological and Cognitive Sciences, Peking University. Written Informed Consent was obtained from all participants before data collection.

### Supplementary Information

Supplementary Information.

## Data Availability

The datasets used and/or analysed during the current study available from the corresponding author on reasonable request.
