# Supplementary material for: Health involvement modulates physician preference in the brain during online health consultation
Source: Sci Rep. 2024 Jan 13;14:1269. doi: 10.1038/s41598-024-51519-4 (PMC10787842; doi:10.1038/s41598-024-51519-4)
Supplement: Supplementary file 1 — Supplementary Information. [file 41598_2024_51519_MOESM1_ESM.pdf]

Supplementary 1

(a)

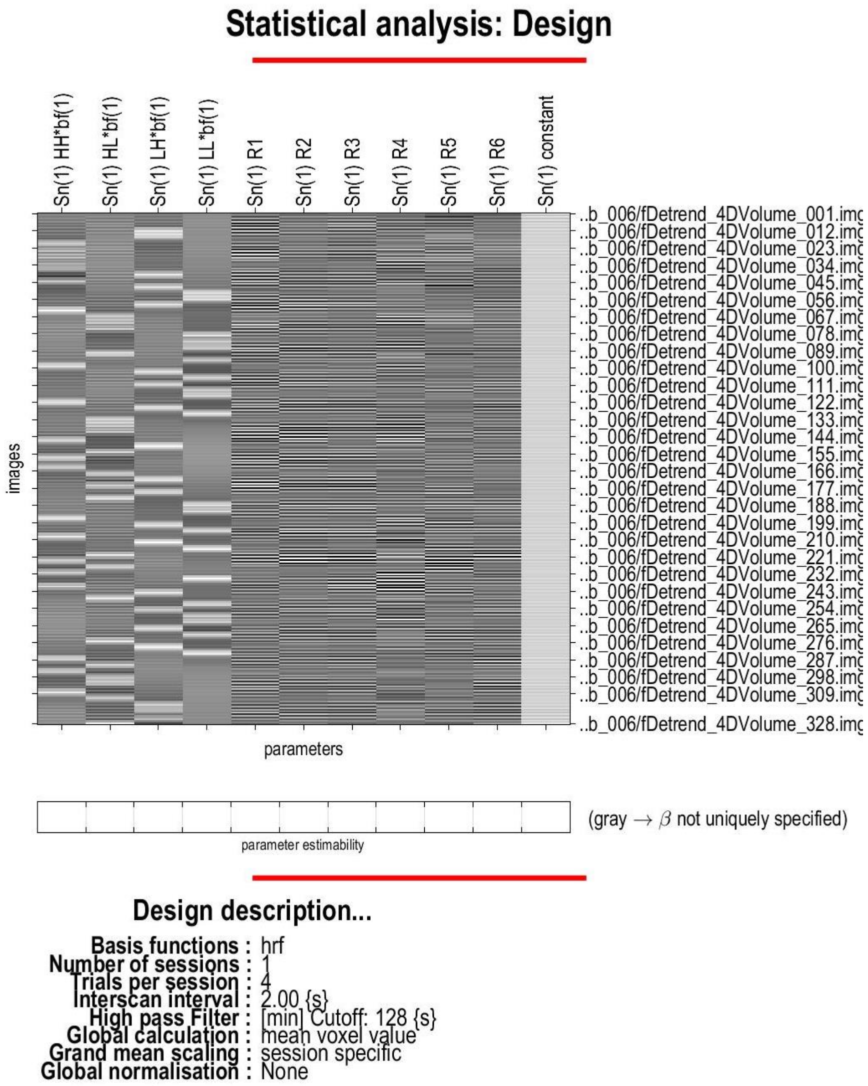

(b)

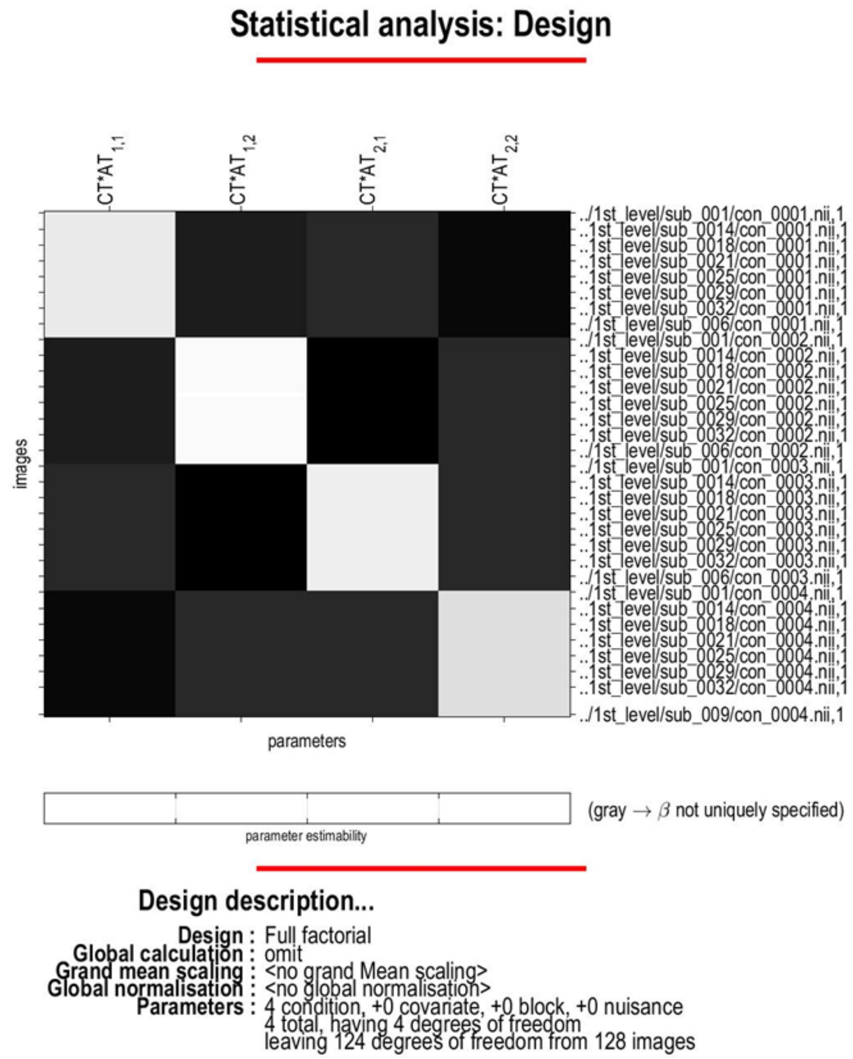

Supplementary 1. Figure S1 Additional experimental parameters and design matrix used in the SPM analysis. Note: (a) additional experimental parameters and design matrix used in Specify 1st-level analysis; (b) additional experimental parameters and design matrix used in Specify 2nd-level analysis.

Supplementary 1. Table S1 Whole brain analysis results with different levels of health involvement

Health Involvement	Brain Region	Hemisphere	Cluster Size	MNI Coordinates x	y	z	T-value
Low Level Health Involvement	**CT contrast: [(Hh + Hl) - (Lh + Ll)**						
	AMYG	L	11	-28	4	-18	4.30
	REC	R	39	2	46	-16	3.99
	IOG	L	53	-28	-94	-10	4.18
	CAL	R	169	10	-88	-2	4.85***
	LING	R	23	10	-70	-10	3.56
	CAL	L	10	-4	-64	16	3.63
	ROL	R	14	38	-18	20	4.12
	**AT contrast: [(Hh + Lh) - (Hl + Ll)]**						
	PUT	L	17	-16	6	-12	4.26
	MCC	R	18	8	-36	40	4.38
	PreCG	L	20	-34	-18	62	4.91
High Level Health Involvement	**CT contrast: [(Hh + Hl) - (Lh + Ll)**						
	-						
	**AT contrast: [(Hh + Lh) - (Hl + Ll)]**						
	-						

Note: Threshold is set to $p < 0.001$, k=10, uncorrected; * Significant at $p < 0.05$ family wise error corrected at the cluster level; AMYG = Amygdala; REC = Rectus; IOG = Occipital_Inf; CAL = Calcarine; LING = Lingual; ROL = Rolandic_Oper; PUT = Putamen; MCC = Cingulate_Mid; PreCG = Precentral;

Supplementary 1. Table S2 Measurement scales

Construct	Items	Sources
Cognitive Trust (CT)	I think the physician will treat his/her work with professionalism and dedication.	1
	Given the physician's description, I do not see any reason to doubt the physician's ability and his preparation for the job.	2
	I am sure the physician will not make the patient's situation more difficult owing to being negligent.	
Affective Trust (AT)	I think the physician will treat me like a friend, and that we will be free to share our thoughts, feelings and hopes with each other.	
	I think the physician will be considerate of and show enough concern for me.	
	If I share my problem with the physician, I think he/she will be enthusiastic and responsible, and will give me useful and considerate advice.	
	I think that, in the consultation process, the physician is willing to consider the interests of both sides and invest with a lot of affection toward the patient to maintain the physician-patient relationship.	
Willingness to Choose (CW)	If I have the chance, I will willingly choose this physician.	3
	Choosing the physician is a foolish idea.	4
	If I have the chance, I will recommend this physician to my family or friends.	5<br>6

References:

1. Mcallister, D. J. Affect- and cognition-based trust as foundations for interpersonal cooperation in organizations. *Academy of Management Journal* **38**, 24-59 (1995).
2. Dong, Y. *Research on factors influencing doctor-patient knowledge transfer based on patient trust*, Zhejiang University, (2010), in Chinese.
3. Pavlou, P. A. Consumer acceptance of electronic commerce: Integrating trust and risk with the technology acceptance model. *International Journal of Electronic Commerce* **7**, 101-134 (2003).
4. Chang, H. H. & Chen, S. W. The impact of online store environment cues on purchase intention Trust and perceived risk as a mediator. *Online Information Review* **32**, 818-841, doi:10.1108/14684520810923953 (2008).
5. Obele, C. C., Duszak, R., Jr., Hawkins, C. M. & Rosenkrantz, A. B. What patients think about their interventional radiologists: assessment using a leading physician ratings website. *Journal Of the American College Of Radiology* **14**, 609-614, doi:10.1016/j.jacr.2016.10.013 (2017).
6. Wang, F. *Research of the violations caused by negative online reviews and the repair*

strategies for the violations: Based on empirical and neurological methods thesis, Beijing University Of Posts and Telecommunications, (2017), in Chinese.
